# Multi-omics comparisons of *p*-aminosalicylic acid (PAS) resistance in *folC* mutated and un-mutated *Mycobacterium tuberculosis* strains

**DOI:** 10.1080/22221751.2019.1568179

**Published:** 2019-02-11

**Authors:** Wenjing Wei, Huimin Yan, Jiao Zhao, Haicheng Li, Zhenyan Li, Huixin Guo, Xuezhi Wang, Ying Zhou, Xiaoli Zhang, Jincheng Zeng, Tao Chen, Lin Zhou

**Affiliations:** a Center for Tuberculosis Control of Guangdong Province, Guangzhou, People’s Republic of China; b Key Laboratory of Translational Medicine of Guangdong, Guangzhou, People’s Republic of China; c Dongguan Key Laboratory of Medical Bioactive Molecular Development and Translational Research, Guangdong Provincial Key Laboratory of Medical Molecular Diagnostics, Guangdong Medical University, Dongguan, People’s Republic of China; d Jinan University, Guangzhou, People’s Republic of China; e School of Stomatology and Medicine, Foshan University, Foshan, People’s Republic of China; f South China Institute of Biomedicine, Guangzhou, People’s Republic of China

**Keywords:** P-Aminosalicylic acid, multi-omics, Mycobacterium tuberculosis, folC

## Abstract

*p*-Aminosalicylic acid (PAS) is an important second-line antibiotic for treating multidrug-resistant tuberculosis (MDR-TB). Due to gastrointestinal disturbance and intolerance, its potent and efficacy in the treatment of extensively drug-resistant (XDR)-TB commonly are poor. Thus, it is important to reveal the mechanism of susceptibility and resistance of *Mycobacterium tuberculosis* (Mtb) to this drug. Herein, we screened and established PAS-resistant (PAS^r^) *folC* mutated and un-mutated Mtb strains, then utilized a multi-omics (genome, proteome, and metabolome) analysis to better characterize the mechanisms of PAS resistance in Mtb. Interestingly, we found that promotion of SAM-dependent methyltransferases and suppression of PAS uptake via inhibiting some drug transport associated membrane proteins were two key pathways for the *folC* mutated strain evolving into the PAS^r^ Mtb strain. However, the *folC* un-mutated strain was resistant to PAS via uptake of exogenous methionine, mitigating the role of inhibitors, and promoting DfrA, ThyA and FolC expression. Beyond these findings, we also found PAS resistance in Mtb might be associated with the increasing phenylalanine metabolism pathway. Collectively, our findings uncovered the differences of resistant mechanism between *folC* mutated and un-mutated Mtb strains resistant to PAS using multi-omics analysis and targeting modulators to these pathways may be effective for treatment of PAS^r^ Mtb strains.

## Introduction

Tuberculosis (TB), caused by the organism *Mycobacterium tuberculosis* (Mtb), is a bacterial disease, infecting approximately one-third of the world’s population [1]. Currently, a standard TB treatment includes a prolonged course of antibiotics lasting from 6 months to 2 years [2], that often results in the development of resistance to one or more antibiotics. Antibiotic-resistant Mtb is a major threat to public health. Clearly, greater efforts are required to improve our understanding of this disease and strengthen overall TB control, in order to curb this problem.

Since 1946, *para*-Aminosalicylic acid (PAS) has been used as a second-line drug for killing Mtb [3]. Shortly before the clinical introduction of PAS, streptomycin as a monotherapeutic drug significantly improved the survival rate of tuberculosis [3]. However, the rapid emergence of streptomycin-resistant Mtb strains posed a threat to this monotherapy strategy for TB infection [3,4]. As PAS is effective against streptomycin-resistant Mtb strains, PAS-based drug resistance combination therapy regimens including streptomycin-PAS-isoniazid treatment regimens, have been widely used [4]. Recently, the World Health Organization drug-resistant tuberculosis (DR-TB) 2016 guidelines reclassified PAS as Group D3 “add-on” drug, while Desai et al., in view of the safety and efficacy of PAS in 250 cases DR-TB patients except for XDR and Category V group, it is recommended to reclassify PAS into Group C rather than Group D3 [5].

Indeed, PAS has been found to dramatically improve cure rates and further reduce the emergence of drug resistance [3,4]. However, PAS is often associated with a high rate of gastrointestinal disturbance which limits its use to the treatment of multi-drug resistant TB [4,6]. Thus, it is important to develop novel strategies to enhance PAS potency, limit adverse reactions and improve treatment success rates. Until recently, the molecular and biochemical mechanisms governing the susceptibility and resistance of Mtb to PAS have not been clearly defined.

It is known that PAS is a selective antimetabolite of the Mtb folate metabolic pathway acting as a structural analog of *para*-aminobenzoic acid (PABA), which is produced from chorismate by the concerted action of aminodeoxychorismate synthase (PabAB) and aminodeoxychorismate lyase (PabC) [7,8]. PAS is sequentially converted to 2′-hydroxy-7,8-dihydropteroate and 2′-hydroxy-7,8-dihydrofolate by enzymes in the Mtb folate metabolic pathway to potently inhibit Mtb dihydrofolate reductase (DHFR) [4,9–12]. A study using transposon mutagenesis identified loss-of-function mutations in *thyA*, encoding a folate-dependent thymidylate synthase, associated with resistance to PAS that were also present in clinical isolates resistant to PAS [13]. In addition, various missense mutations in *folC* encoding dihydrofolate synthase and over-expression of RibD can confer Mtb resistance to PAS [12,14], while, deleting *pabC* caused increased susceptibility of Mtb to PAS [15].

Another study showed that PAS can be converted to N, N-dimethyl PAS species without anti-tubercular activity in Mtb cells [8]. Since the addition of methionine can potentially enhance the ability of Mtb to methylate PAS by increasing S-adenosylmethionine (SAM) abundance, it is possible that methionine promotes inactivation of PAS through N,N-dimethylation by an unidentified methyltransferase. However, Howe *et al.* have demonstrated that methionine-mediated antagonism of anti-folate drugs occurs through the sustained production of folate precursors [16]. In addition, they found that intracellular biotin confers intrinsic PAS resistance in a methionine-independent manner [16].

Therefore, new approaches are required to better characterize the mechanisms relating to drug resistance [17,18]. With all this in mind, we used a multi-omics approach, integrating genomic, comparative proteomic and metabolomics to predict new PAS resistance mechanisms in two well-described laboratory PAS-resistant Mtb strains (*folC* mutated PAS^r^1 Mtb strains, *folC* un-mutated PAS^r^2 Mtb strains). As a result, our findings reveal that these two PAS-resistant Mtb strains have different resistance mechanisms. *folC* mutated strains evolved to PAS resistance via two pathways: (1) reducing bioactivation of PAS by increasing the abundance of SAM-dependent methyltranferases and *folC* mutation; (2) reducing the PAS uptake via decreasing expression of membrane proteins, especially ABC transporters. Moreover, we also found an increasing phenylalanine metabolism pathway in the PAS^r^*folC* mutated Mtb strains. Different from *folC* mutated strains, the *folC* unmutated strains were found to harbour PAS resistance via retaining methionine and mitigating the impact of target inhibitor, viz., overexpression of DfrA, ThyA and FolC.

## Results

### Establishment of *folC* mutated and un-mutated PAS^r^ Mtb strains

**Figure 1.**
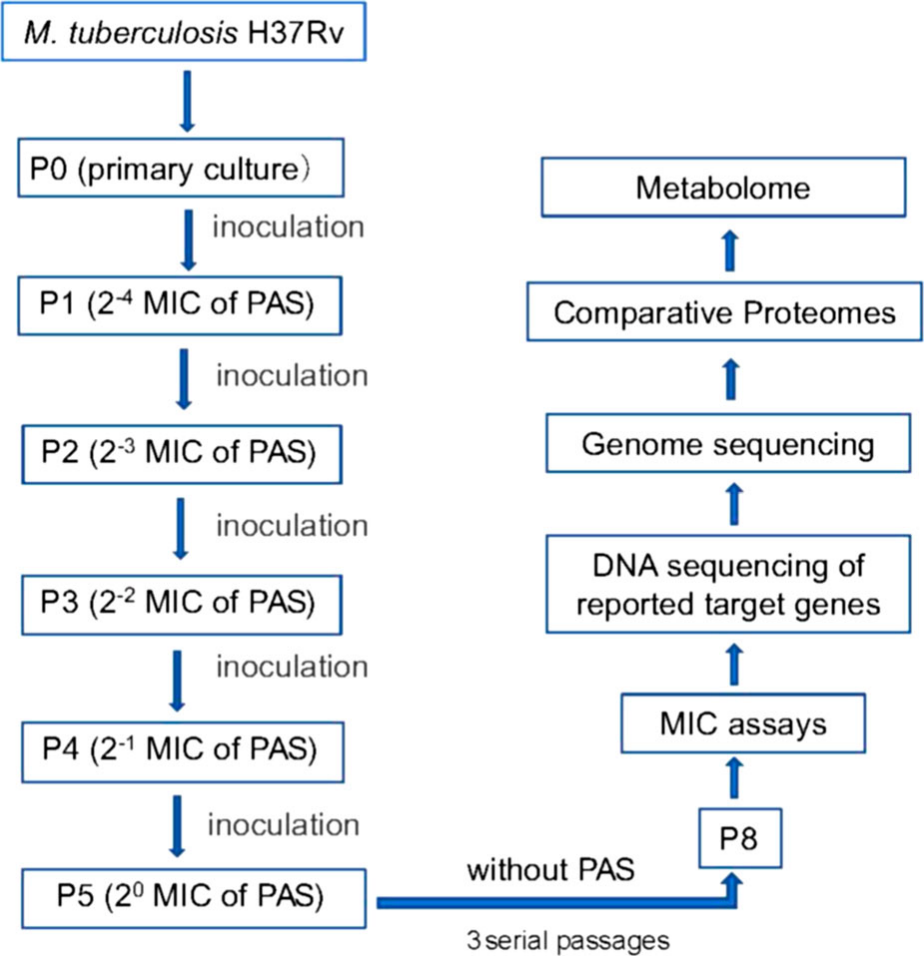
Outline of experimental scheme in this study. *M. tuberculosis* H37Rv strain was cultured on Löwenstein-Jensen (LJ) medium and processed by amplification culture named as passage 0 (P0) strains, which were selected for preparing PAS-resistant strains. Concentration gradients (2^−4^, 2^−3^, 2^−2^, 2^−1^, 2^0^) of PAS based on the critical concentration (1.0 mg/L PAS) contained LJ medium were prepared, and P0 strains (1 MCF) were cultured in LJ medium (2^−4^ PAS concentration) for approximately 4 weeks named as passage 1 (P1). Repeat this step till passage 4 (P5) strains, which met to the WHO criteria for PAS-resistant strains, and then the resistant strains were verified by the appropriate concentrations of drug used in the drug-susceptibility test. The resistant strains were continuously cultured to passage 8 (P8) without PAS, and then analysed by the drug-susceptibility test. 218 single colonies were randomly selected and cultured in liquid culture for the extraction of genomic DNA and testing PAS MICs of Mtb strains. Then single colonies were assessed by targeted sequencing of PCR amplicons of genes associated with PAS resistance. The primers used in the targeted sequencing are described in Supplemental Table S8. Two strains, one folC mutant and one non-folC mutant are identified and subjected to full genome resequencing, proteomic and metabolomic analysis.

To obtain PAS^r^ Mtb strains, wild type H37Rv Mtb strains were treated with PAS according to the route of Figure 1. Finally, 109 PAS^r^ Mtb clones (MIC > 2 μg/ml) were randomly selected for further sequencing to screen mutation of PAS^r^ associated genes *folC, dfrA*, *thyA*, *folP1* and *ribD*. Results showed 84 of 109 PAS^r^ strains (named PAS^r^ 1 group) only with mutation of *folC* gene (448A→C, S150R) after PAS treatment. However, we did not find any mutations on *dfrA*, *thyA*, *folP1* and *ribD* gene both on other 25 PAS^r^ strains (named PAS^r^ 2 group) and the PAS un-treated parental strains (named Control group) (Supplemental Table S1).

**Figure 2.**
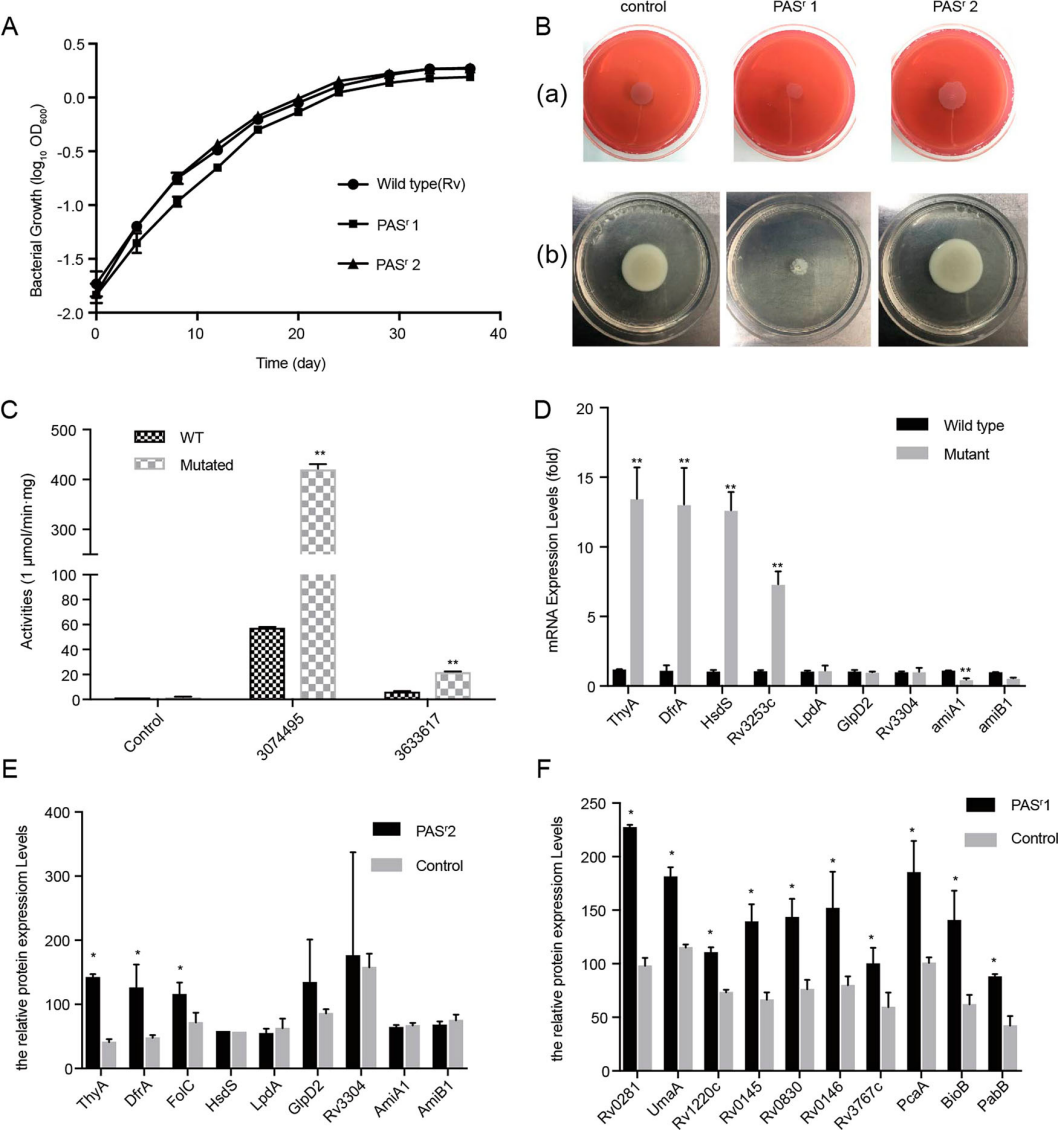
Characterization of PAS-resistant Mtb used in this study. (A) Growth of *M. tuberculosis* H37Rv, PAS^r^ 1 and PAS^r^ 2 strains in 7H9 + ADC liquid media for 37 days. The average of two biological replicates is shown. (B) Phenotypic analysis of *M. tuberculosis* strains. Wild-type strain (control) was compared to *folC* mutated PAS^r^ strains (PAS^r^1) and *folC* unmutated PAS^r^ strains (PAS^r^2) for the following phenotypes: (a) Colony morphology, (b) Sliding motility. (C) IGR SNPs alter the expression level of downstream genes. Two IGRs on the expression of lacZ in *M. smegmatis* were examined by performing β-D-galactosidase activity assays on IGR-lacZ constructs. The IGR sequences, with or without the SNPs (3074495 G→A, located in between *thyA* and *Rv2765* and 3633617 C→T, located in between *Rv3253c* and *Rv325*4) identified in this study, were inserted into the pSD5B mycobacterial shutter vector that contains a promoterless lacZ reporter gene immediately downstream of the cloning site and β-D-galactosidase activity was assayed. Error bars represent the s.d. calculated from three separate measurements. (D) Semi-quantitative RT-PCR was used to examine the expression of the IGR downstream genes. *thyA*, *drfA* and *hsdS* are located in the downstream of 3074495 SNP, Rv3253c is located in in the downstream of 3633617 SNP and *lpdA*, *glpD2*, *Rv3304*, *amiA1*, *amiB1* are located in the downstream of VNTR 3690 locus. Data were analysed using the ΔΔC_T_ method with the *M. tuberculosis* reference gene *sigA* as the control. Data were mean values ± SD from independent biological samples. ** represents *P* value < 0.01. (E) iTRAQ was used to examine the expression of the IGR downstream genes in PAS^r^2 Mtb. * represents *P* value < 0.05. (F) SAM-dependent methyltransferases (AdoMet-MT) and some folic acid synthesis-related proteins with significant differences were identified by iTRAQ in PAS^r^1 Mtb compared to the parental strain. * represents *P* value < 0.05.

In order to verify whether the mutation was stable, we cultured the PAS^r^ Mtb strains for continuous three passages without PAS stimulation and found that the *folC* mutation and resistance level to PAS were not changed (data not shown), indicating that the PAS^r^ Mtb strains have been successfully induced and established. Obviously, we also found the *folC* mutated and un-mutated PAS^r^ Mtb strains had different prevalence and growth characteristics, especially in 79.2% (84/109) PAS^r^1 strains (*folC* mutated) comparatively having the largest reduction in growth with higher resistance to PAS (>256 μg/ml) compared with the PAS^r^ 2 strains (*folC* un-mutated, 4 μg/ml) (Figure 2(A,B)), indicating these PAS^r^ strains may have different drug-resistant mechanisms. In addition, these two strains were not cross-resistant to other anti-TB drugs except PAS (Supplemental Table S2). Therefore, we have successfully established stable *folC* mutated and un-mutated PAS^r^ Mtb strains.

### Whole-genome sequence of *folC* mutated and un-mutated PAS^r^ Mtb strains

To reveal the difference mechanism of PAS resistance among *folC* mutated PAS^r^1 Mtb strains, *folC* un-mutated PAS^r^2 Mtb strains and PAS un-treated parental control strains, whole genome sequencing was performed. Results showed that apart from *folC* mutant, there was only a non-synonymous in *esxK* (140G→A) of PAS^r^1 strains (Supplemental Table S3). Notably, a number of mutations including two intergenic regions (IGR) single nucleotide polymorphisms (SNPs) (3074495 G→A, located in between *thyA* and *Rv2765* and 3633617 C→T, located in between *Rv3253c* and *Rv325*4) and an insertion mutation of variable number of tandem repeat (VNTR) 3690 locus can be found in PAS^r^2 strain (Supplemental Table S3).

Previous study has been reported that IGR SNPs may affect the expression levels of flanking genes [19]. To verify the impacts of these IGR SNPs, we designed promoterless *lacZ* gene fusion constructs containing the intergenic sequence between *thyA* and *Rv2765*, and between *Rv3253c* and *Rv325*4, and introduced them into *M. smegmatis*, a saprophyte extensively employed as a surrogate model for Mtb [20], and analysed *lacZ* gene activity. Our results revealed that the *Mycobacterium smegmatis* strains with IGR SNP in *thyA*–*Rv2765* (3074495 G→A) and *Rv3253c*–*Rv325*4 (3633617 C→T) showed 7.3-fold and 3.4-fold higher β-galactosidase activity than IGR un-mutated control *M. smegmatis* strains, respectively (*P* < 0.05) (Figure 2(C)). When a negative control construct (promoterless *lacZ* gene) was introduced, no β-galactosidase activity was detected. Additionally, Parvez et al., previously reported that the copy number of VNTR 3690, located in the intergenic region between *Rv3304* and *Rv3303c* (encoding *gplD2* and *lpdA* genes, respectively), had an effect on the transcription of *lpdA* [21]. These results strongly suggest that the SNPs in these drug resistance–associated IGRs would lead to regulation of the downstream genes in Mtb strains. We verified expression of several downstream genes of the above two SNP sites in PAS^r^ 2 strains by qRT-PCR analysis. Among these genes, *thyA*, *drfA*, *hsdS* and *Rv3253c* in the PAS^r^ 2 strain were significantly increased compared to the parental strain, respectively (*P* < 0.01) (Figure 2(D)).

### Global protein expression profiling comparisons of *folC* mutated and un-mutated PAS^r^ Mtb strains

In order to further investigate the changes in protein levels of the strain caused by PAS resistance, we performed global protein expression profiling on the *folC* mutated and un-mutated PAS^r^ Mtb strains and their parental strains. With a highly conservative threshold, in total, 2341 proteins were identified. In PAS^r^1 strains, compared to its parent strains, a total of 361 proteins expression differences were greater than 1.5-fold (*P* < 0.05), including 290 down-regulated proteins and 71 up-regulated proteins, while in PAS^r^2 strains, 10 down-regulated proteins and 28 up-regulated proteins expression differences were greater than 1.5-fold (Supplementary Table S4). Meanwhile, *thyA* and *dfrA* mRNA expression, which proteins were up-regulated, also increase in PAS^r^2 strains, while VNTR 3890 mRNA expression, which proteins were down-regulated, had no significant changes between PAS^r^2 strains and their parental strains (Figure 2(D,E)). Interestingly, there were no changes of these proteins in *folC* mutated PAS^r^1 strains, while many S-adenosyl-L-methionine (SAM)-dependent methyltransferases (AdoMet-MT) and two proteins, PabB and BioB, were overexpressed in PAS^r^1 strains (Figure 2(F)).

**Figure 3.**
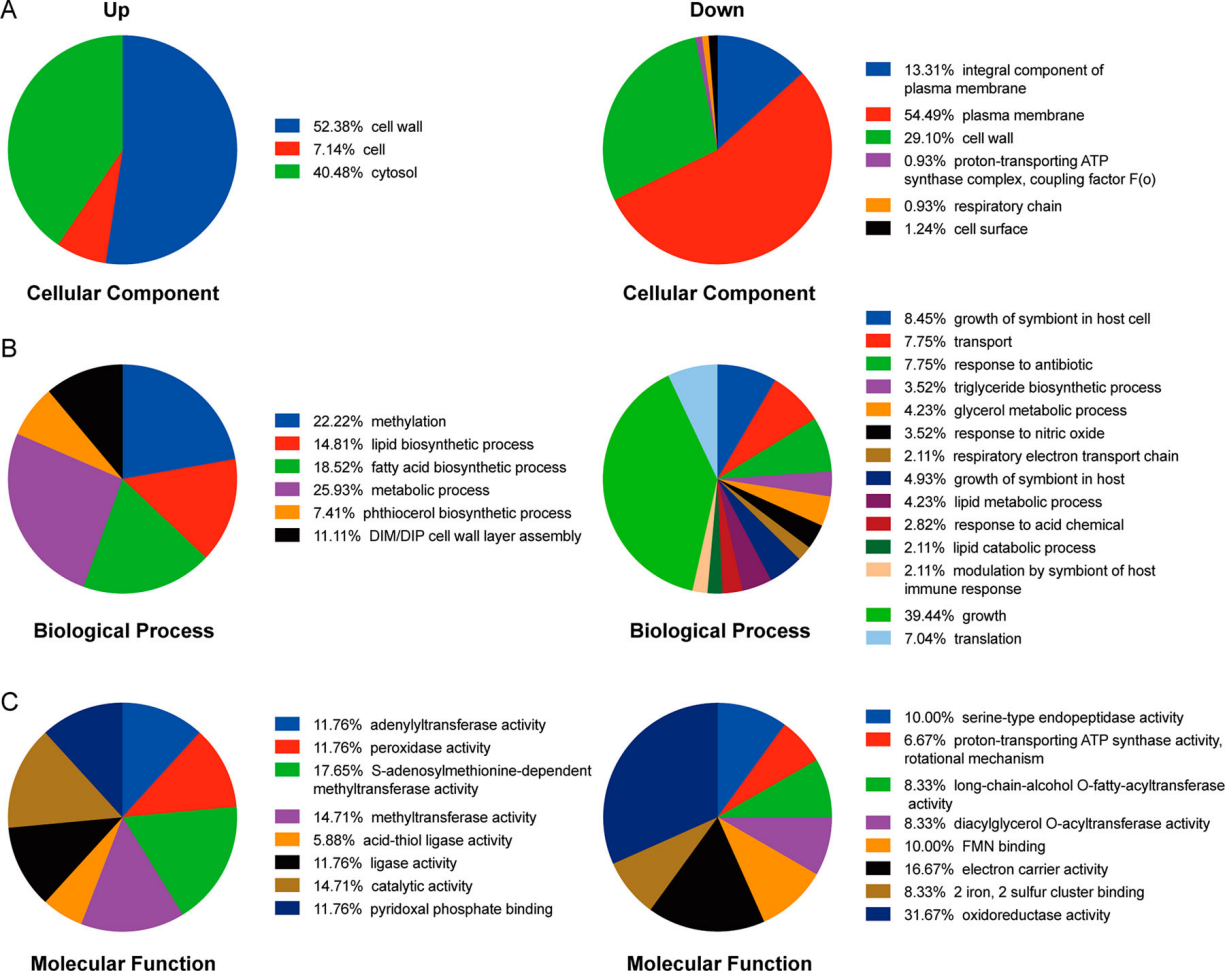
Gene Ontology categories for the differentially expressed proteins in PAS^r^1 strain compared with those in its parent strain using DAVID analysis. (A) Functional classification of the differentially expressed proteins in the cellular component that increased and decreased, respectively. (B) Functional classification of the differentially expressed proteins in the biological processes that increased and decreased, respectively. (C) Functional classification of the differentially expressed proteins in the molecular functions that increased and decreased, respectively.

Additionally, to further investigate the biological process that mediated by the above differential proteins from PAS^r^1 strains and PAS^r^2 strains, the Gene Ontology (GO) categories were evaluated using DAVID online software (Supplemental Table S4). As a result, in PAS^r^1 strains, 178 down-regulated proteins (61.6%) were associated with plasma membrane, 43 down-regulated proteins (14.8%) related to an integral component of plasma membrane, and 94 down-regulated proteins located on the bacterial cell wall (Figure 3(A)). Notably, the biological transport process was also significantly enriched (Figure 3(B)). According to previous reports, Mtb encodes at least 46 putative drug efflux, and in this study, we found 6 efflux pumps (Rv1218c, Rv1458c, Rv1819c, DrrA, DrrB, MmpL3 and PstB) were observably decreased in PAS^r^ 1 strains (Supplemental Table S4). These results suggested that the down-regulated membrane proteins may be associated with decreased PAS uptake.

As mentioned above, PAS^r^1 strains grew to a lower yield than the parental control strains (Figure 2(A)). It is noteworthy that 56 down-regulated proteins associated with the PAS^r^1 strains growth process indicating that these changes may be related to impaired growth (Figure 3(A)). Additionally, 17.65%, 14.71% and 14.71% of up-regulated proteins mediated S-adenosylmethionine-dependent methyltransferase activity, methyltransferase activity, pyridoxal phosphate binding activity in PAS^r^1 strains, respectively. Meanwhile, 19 of 290, 10 of 290 down-regulated proteins mediated oxidoreductase activity and electron carrier activity, respectively (Figure 3(C)). Moreover, 5 down-regulated proteins (Rv0221, Tgs1, Tgs2, Rv3087 and Rv2285) involved in long-chain-alcohol O-fatty-acyltransferase activity, and 4 down-regulated proteins (AtpB, AtoF, AtpH and AtpG) involved in proton-transporting ATP synthase activity were enriched, which ultimately led to a decrease in the bacterial energy generation system. (Figure 3(C)). These results suggest that *folC* mutation-mediated PAS resistance may be associated with these processes.

### Phenylalanine, tryptophan and histidine metabolism pathways were enriched by up-regulated proteins in *folC* mutated PAS^r^ Mtb strains

**Figure 4.**
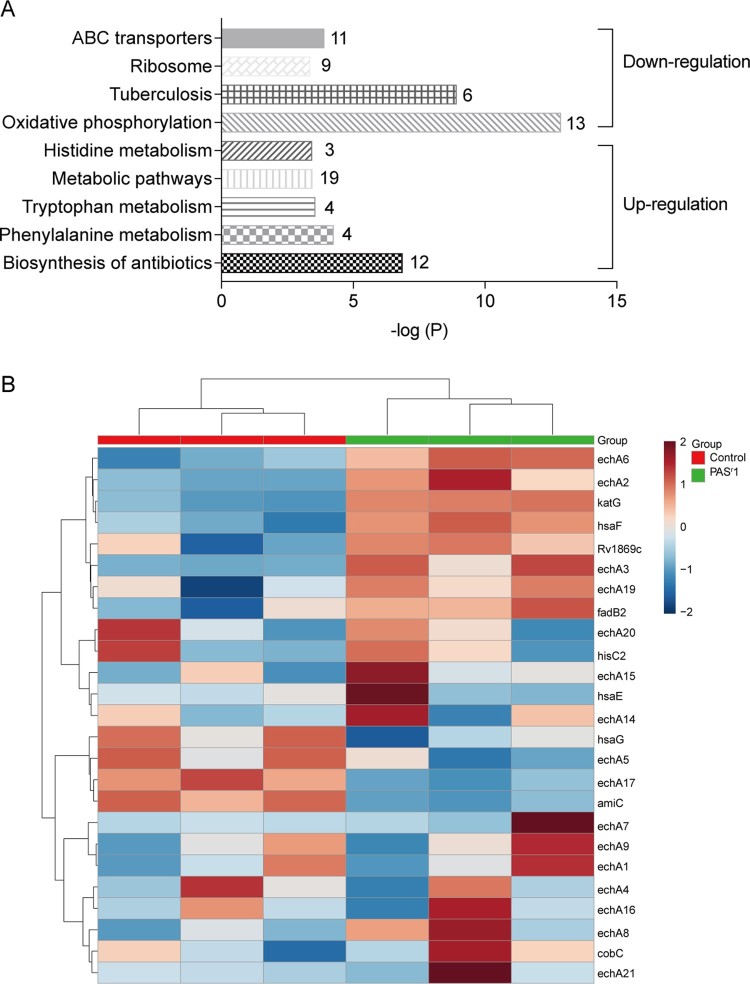
Histogram displaying KEGG pathway of the differentially expressed proteins in PAS^r^ 1 strain according to the *P* value. (A) The upregulated and downregulated pathway were marked. Each related gene numbers is shown on the right of the bars. (B) Heat map showing the average expression level of proteins related to phenylalanine metabolism in in PAS^r^1 Mtb compared to its parental strain.

The KEGG pathways analysis using significantly altered proteins in *folC* mutated PAS^r^ 1 Mtb strains showed that ATP-binding cassette (ABC) transporters, ribosome, tuberculosis and oxidative phosphorylation pathways were enriched by down-regulated proteins, while antibiotics, phenylalanine metabolism, tryptophan metabolism and histidine metabolism pathways were enriched by up-regulated proteins (Figure 4(A,B), Supplemental Table S4). However, there was no pathways were enriched by using 10 down-regulated or 28 up-regulated proteins in *folC* un-mutated PAS^r^ 2 Mtb strains. *folC* encodes the enzyme responsible for folate polyglutamylation. Several recent studies found that *folC* mutations mapped within positions corresponding to substrate and nucleoside binding domains result a reduced bioactivation of PAS [10,12,14]. However, the exact mechanism of how *folC* mutations regulate bioactivation of PAS is unknown. Herein, our results suggest that phenylalanine, tryptophan and histidine metabolism pathways may be involved in the regulation of bioactivation of PAS in *folC* mutated PAS^r^ 1 Mtb strains.

### Phenylalanine metabolism pathways was significantly enriched by up-regulated intracellular metabolites in *folC* mutated PAS^r^ Mtb strains

**Figure 6.**
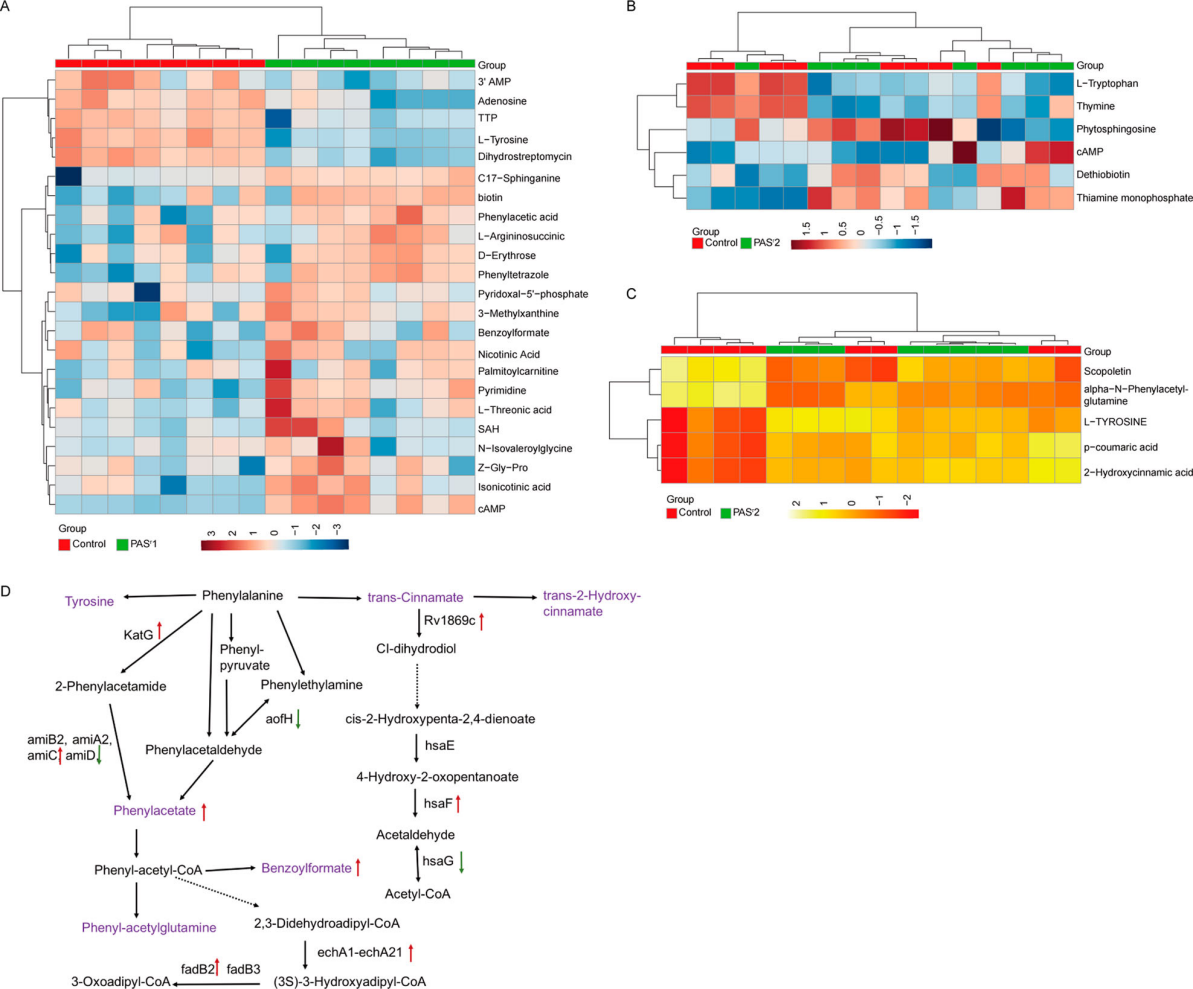
The significantly alterated metabolites related to phenylalanine metabolism pathways were observed in intracellular and extracelluar metabolites of PAS^r^ Mtb strains. (A) The intracellular metabolites in PAS^r^ 1 Mtb strain. (B) The intracellular metabolites in PAS^r^ 2 Mtb strain. (C) The extracellular metabolites in PAS^r^ 1 Mtb strain. (D) A schematic summary of the metabolic and proteomic changes involved in phenylalanine metabolism in PAS^r^ Mtb strains. The purple characters represent the different metabolites found in the PAS^r^ Mtb strain. The red arrows indicate significantly increased proteins, while the green arrows indicate significantly decreased proteins in PAS^r^ 1 Mtb strain compared to its parental strain.

To investigate the metabolomics level in the *folC* mutated and un-mutated PAS^r^ Mtb strains, the intracellular and extracellular metabolites were evaluated by liquid chromatography coupled with tandem mass spectrometry (LC–MS) and analysed by multi- and univariate analyses [22]. Results showed that the total variance of metabolomes data in *folC* mutated and un-mutated PAS^r^ Mtb strains with their corresponding parental control strains was 63.8% (*folC* mutated PAS^r^1 Mtb strains) and 47.1% (*folC* un-mutated PAS^r^2 Mtb strains) by principal component analysis (PCA), respectively (Figure 5(A,B)). Two-dimensional (2D)-PCA score plot revealed that both PAS^r^1 (42.7% of the variance) and PAS^r^2 (28.8% of the variance) Mtb strains can be distinguished from their corresponding parental strains based on the first principal components. Subsequently, partial least squares-discriminant analysis (PLS-DA) was used to maximize the separation and identify additional metabolites (Figure 5(C,D)). Then, potential metabolites were selected based on the VIP score (>1) after univariate analysis (Supplemental Table S5). First, we observed that antifolate-generated thymine starvation occurred in *folC* mutated PAS^r^1 Mtb strains with a 2-fold decrease of thymidine-5’-triphosphate (TTP), and in *folC* un-mutated PAS^r^2 Mtb strains with reduction of thymine (Figure 6(A,B), Supplemental Table S5), which is consistent with previous reports about PAS-resistant Mtb [4]. Notably, only phenylalanine metabolism pathway was significantly enriched by up-regulated intracellular metabolites (Supplemental Table S5), which is consistent with the enriched results by up-regulated proteins such as HsaF, KatG, EchA3 and EchA6, in *folC* mutated PAS^r^1 Mtb strains (Figure 4(B), Supplemental Table S4). In addition, S-Adenosyl-L-homocysteine (SAH), a by-product of SAM-dependent methyltransferase reactions and biotin were also significantly increased in *folC* mutated PAS^r^1 Mtb strains (Figure 6(A)). Moreover, glycerophospholipid, purine and inositol phosphate metabolism pathways were significantly enriched by down-regulated intracellular metabolites (Supplementary Table S5). Figure 5.PCA score plot and PLS-DA score plot generated using MetaboAnalyst based on intracellular and extracelluar metabolites of PAS^r^ strains in positive mode compared with their parent strain, respectively. (A–D) Intracelluar metabolites, (E–H) Extracelluar metabolites. (A, B, E, F) A two-dimensional PCA score plot indicating the natural grouping and differentiation of the individual samples into samples into the PAS^r^ strains and the control strain, due to variation in their metabolic profiles. (C, D, G, H) Two-dimensional PLS-DA score plot. Predictive component 1 and component 2 can differentiate the PAS^r^ strains and the control strain.

**Figure:**
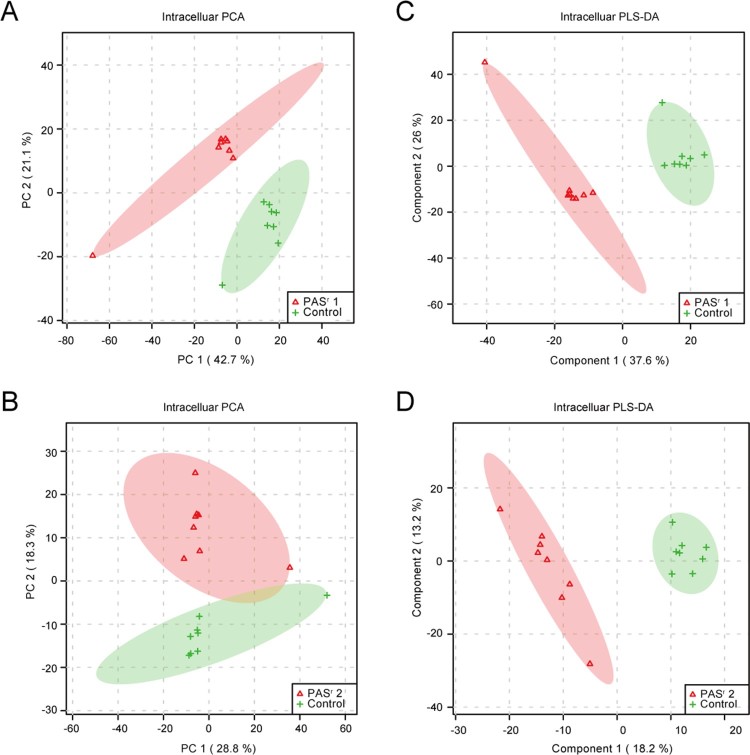

**Figure:**
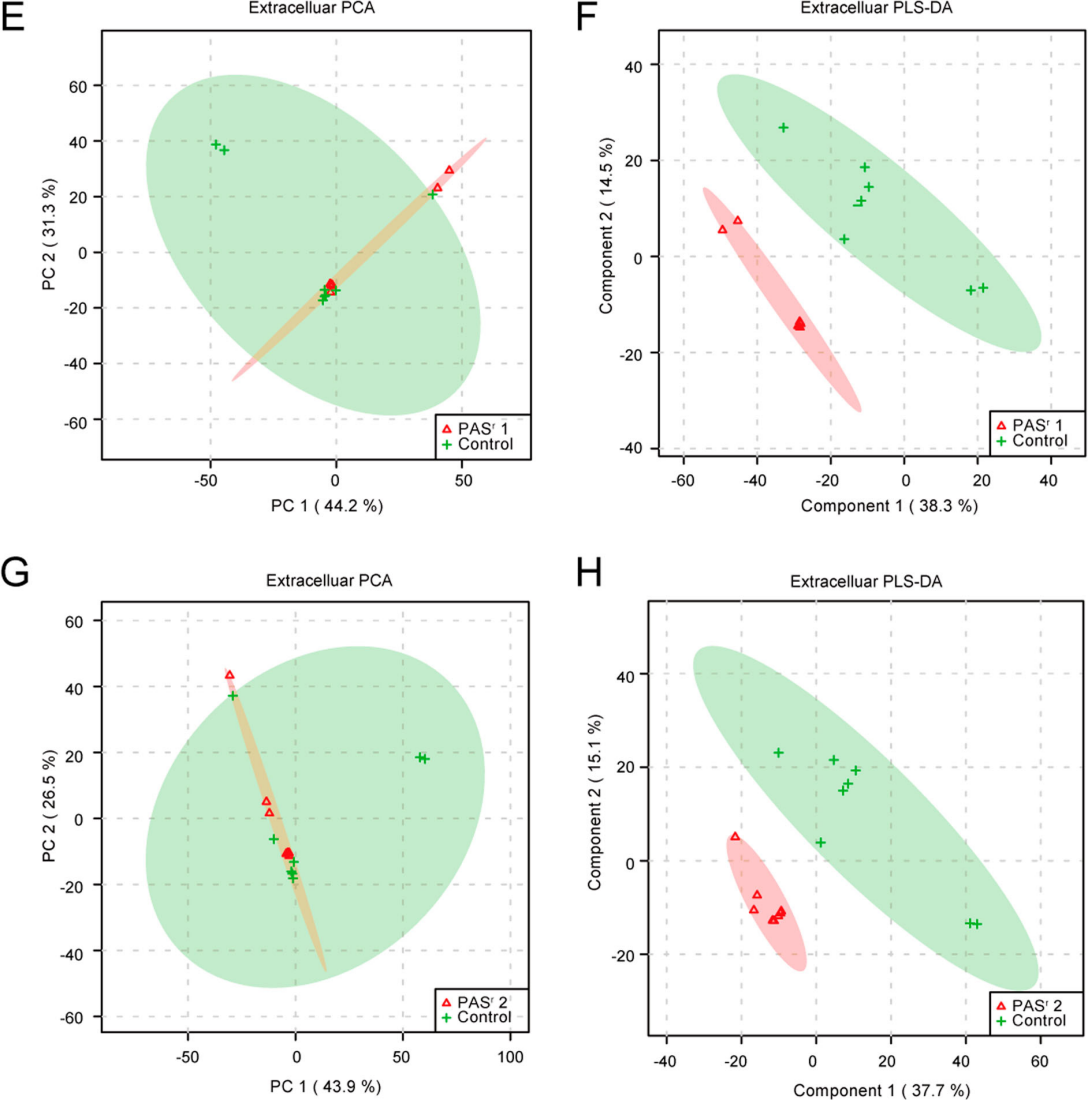

Furthermore, we assessed the extracellular metabolites using culture supernatant of *folC* mutated and un-mutated PAS^r^ Mtb strains with their corresponding parental control strains after PCA and PLS-DA analysis (Figure 5(E,F)). Finally, 79 and 106 putative metabolites were identified in *folC* mutated PAS^r^ 1 Mtb strains and un-mutated PAS^r^ 2 Mtb strains, respectively (Supplementary Table S6). Consistent with change of intracellular metabolites, thymidine was decreased both on culture supernatant of PAS^r^1 and PAS^r^2 Mtb strains (Supplemental Table S7). Notably, methionine, which can antagonize PAS activity via affecting folate precursor biosynthesis pathway was also significantly decreased on culture supernatant of PAS^r^2 Mtb strains (Supplemental Table S7). KEGG pathways analysis showed that cysteine and methionine metabolism, biosynthesis of unsaturated fatty acids and linoleic acid metabolism were significantly enriched by up-regulated extracellular metabolites, while phenylalanine metabolism was significantly enriched by down-regulated extracellular metabolites in *folC* mutated PAS^r^1 Mtb strains (Supplemental Table S6). In *folC* un-mutated PAS^r^ 2 Mtb strains, aminoacyl-tRNA biosynthesis, metabolic pathways, ABC transporters and phenylalanine metabolism were significantly enriched by down-regulated extracellular metabolites (Supplemental Table S6). Interestingly, no significant changes in the proteins and metabolites associated with phenylalanine metabolism were detected within the PAS^r^2 strain, while the significantly reduced metabolites associated with phenylalanine metabolism were detected outside the cells (Figure 6(C)). In brief, our proteomic and metabonomic results indicated phenylalanine metabolism might be associated with PAS^r^ resistance in Mtb (Figure 6(D)).

## Discussion

In this study, we used the increasing concentrations of PAS *in vitro* to produce two different PAS-resistant strains (*folC* mutated and un-mutated) and use an integrated genomic-proteomic-metabonomic approach to compare the differentially expressed proteins and metabolites of PAS-resistant Mtb and their parent strain.

### *folC* is the most direct and predominant target of PAS in Mtb

To detect gene mutations directly caused by PAS, we established a PAS-drug resistance model of Mtb *in vitro* by a continuous drug concentration gradient induction (Figure 1, Supplemental Experimental Procedures). Different from the method induced by high concentration drugs [10], this method can better simulate the accumulation of drugs in the body. Interestingly, among the reported PAS^r^ associated genes, we only found there was an about 77% mutation rate in *folC* (Supplemental Table S1). These results suggested that *folC* mutation was the most predominant domains on PAS^r^ Mtb strains, consistent with Zhang *et al.,* findings that 34.8% (72/208) of *folC* mutation have been found in isolated clinical PAS^r^ Mtb strains [14].

It is known that FolC can activate PAS through forming the dihydrofolate (H_2_Pte-Glu) analog hydroxyl dihydrofolate (H_2_PtePAS-Glu). Crystal structures show that a four-helix bundle (α1–α2/α4–α5), which the FolC mutation (Ser150Arg) was located in, plays an important role in the interaction with hydroxyl dihydropteroate (H_2_PtePAS), an analog of H_2_Pte, indicating that this mutant may affect the efficiency of H_2_PtePAS glutamination. This mutant was also observed in isolated clinical PAS^r^ Mtb strains [14]. Compared to laboratory isolated PAS^r^ Mtb strains, there were also several different *folC* mutations observed in clinical PAS^r^ Mtb strains. For example, *Zhang* et al., has reported that five specific residues (positions 40, 43, 49, 150, and 153) accounted for 94.4% (68/72) of the *folC* mutant isolated from patients, while no mutations except Ser150 were detected in the spontaneous mutants in this study. *Zhao et al.,* found many laboratory *folC* mutant isolates using a method induced by high concentration of PAS and the mutation of Ser150 in *folC* was also included [10]. This discrepancy in mutational stability may be related to different strain backgrounds, as well as their difference in growth *in vivo* versus *in vitro*.

### *folC* mutated and un-mutated PAS^r^ Mtb strains have different drug-resistant mechanisms

Compared to *fol*C mutated PAS^r^ Mtb, the un-mutated Mtb contains more genetic mutations in the genome, including several IGR mutations to regulate the expression of proteins related to PAS-resistance, such as DfrA (Figure 2, Supplemental Table S3 and Supplemental Table S4). Zheng et al. previously reported that overexpression of *dfrA* was sufficient to confer resistance to PAS in Mtb [12]. Although there were less genomic changes of *fol*C mutated Mtb, proteomic results showed many AdoMet-MTs, pabB and bioB were overexpressed (Figure 2 and Supplemental Table S4). AdoMet-MT can convert PAS to an inactive status by N,N-methylation [8]. PabB is an essential enzyme required to convert chorismate to PABA in Mtb [23–25]. BioB is a radical SAM-dependent enzyme required for the final step in the synthesis of biotin and the intracellular biotin confers intrinsic PAS resistance. Metabolomic results further showed S-Adenosyl-L-homocysteine (SAH) and biotin were significantly increased in *folC* mutated PAS^r^1 Mtb strains (Figure 6 and Supplemental Table S5). These results suggest that PAS^r^1 strain is more likely to inactivate PAS through N, N-dimethylation by SAM methyltransferases and at the same time, increase the intracellular biotin to confer intrinsic PAS resistance in a methionine-independent manner.

In addition, we found several ABC transporters and many other membrane proteins were significantly decreased in *fol*C mutated PAS^r^ Mtb (Figure 3 and Supplemental Table S4). Efflux pumps play a major role in bacterial drug resistance. ABC transporters (one family of efflux pump), which consists of both exporters and importers, are conserved from bacteria to humans, and their activity are significantly associated with the clinical phenotype, toxic events, and drug disposition [26,27]. ABC exporters are responsible for transporting a variety of substrates such as antibiotics, proteins and lipids, while ABC importers transport a broad range of substrates including amino acids, peptides, sugars, metals and other metabolites [28]. Wang et al. found ABC transporter, Rv1217c-Rv1218c to be overexpressed in clinical isolates from MDR-TB patients [29]. Intracellular PAS is excluded by the efflux antiporter TAP of Mtb. Recently, Ramón-García et al. found that a major facilitator superfamily drug efflux pump, Tap (Rv1258c), conferred resistance to PAS on *M. bovis* BCG [30]. However, our results in here showed that the *folC* mutated PAS^r^ 1 Mtb strains may not efflux PAS through ABC transporters, but reduce the PAS uptake by the down-regulated membrane proteins to produce resistance to PAS.

There is another interest finding in PAS^r^ 1 Mtb, viz., the phenylalanine metabolism pathway was enriched at the level of both proteome and metabolome (Figure 4, Figure 6, Supplemental Tables S4, S5 and S6). Recently, Patel et al. found phenylalanine-derived (Z)-5-arylmethylidene rhodanines as anti-methicillin-resistant *Staphylococcus aureus* (MRSA) compounds [31]. Another report has shown phenylalanine metabolism can affect natural synthesis of penicillin G in *Penicillium chrysogenum* by offering the side chain precursor phenylacetate [32]. Thus, it can be seen that phenylalanine metabolism is related to the drug resistance of pathogenic bacteria, but how does it affect the drug resistance of Mtb need further study.

**Figure 7.**
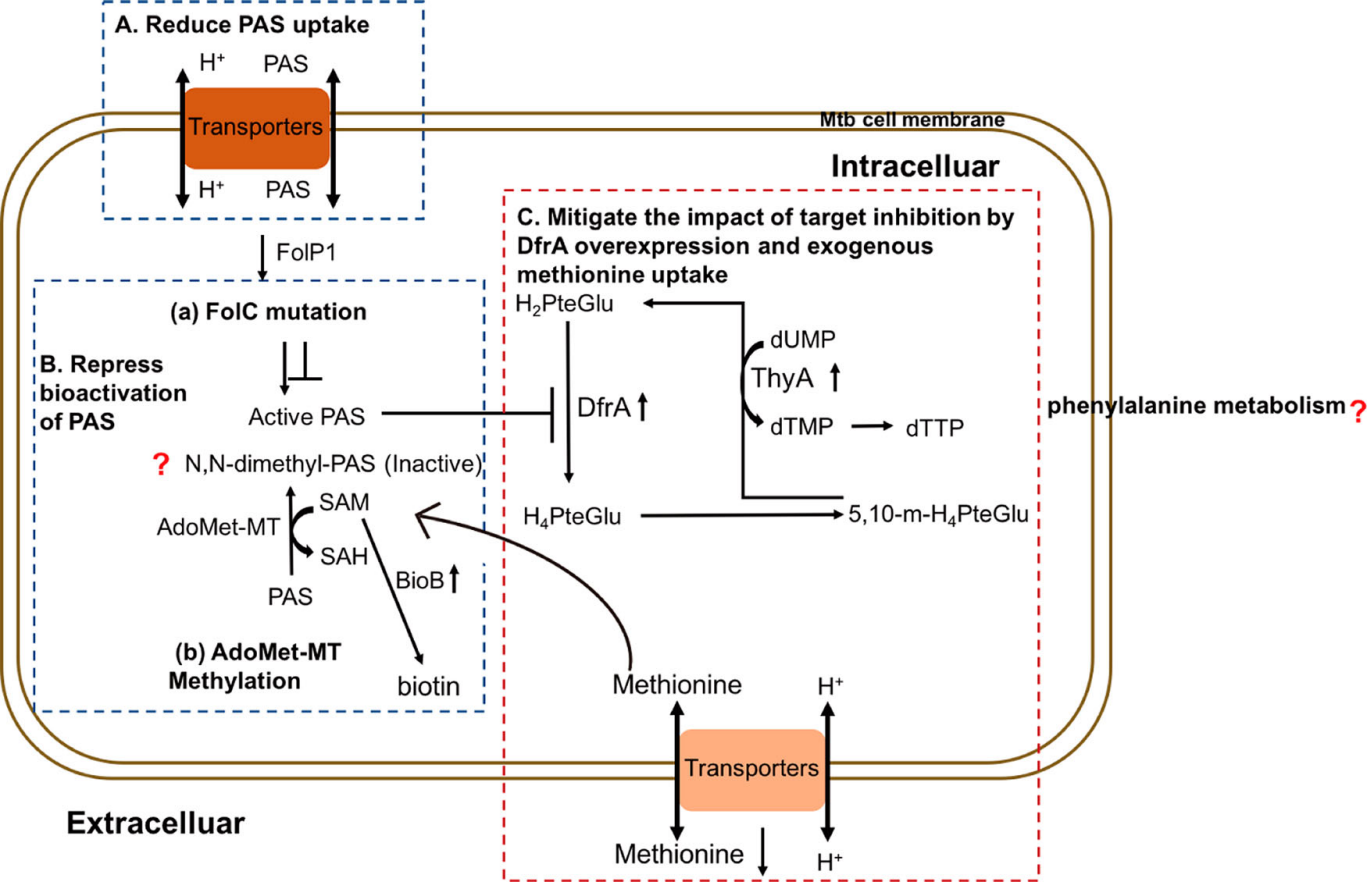
Mechanisms of PAS resistance in this study. Three characterized mechanisms relative to this study are shown. (A) Reduce PAS uptake. The PAS uptake is reduced in PAS^r^1 strain via the decreasing membrane proteins, especially ABC transporters. (B) Repress bioactivation of PAS. PAS^r^1 strain can diminish bioactivation of PAS by increasing the abundance of SAM-dependent methyltranferases and FolC mutation. (C) Overexpression of DfrA and ingesting exogenous methionine. In PAS^r^2 strain, inhibition of dihydrofolate reduction can be negated by DfrA overexpression and exogenous methionine uptake. Blue dashed doxes represent PAS^r^1 and red dashed dox represent PAS^r^2 strain.

In summary, muti-omics is an uprising approach in pathogen and infectious disease research. Using this revolutionary method, we found *folC* mutated and un-mutated Mtb have different PAS-resistant mechanisms and a schematic summary of the mechanisms is given in Figure 7. PAS^r^1 strain evolved into a PAS-resistant Mtb via reducing bioactivation of PAS and the PAS uptake, while PAS^r^2 strain has been shown to confer PAS resistance via overexpression of DfrA and limiting extracellular accumulation of methionine. Currently, we also use genetic and biochemical analysis, such as *DfrA* gene replacement and transport inhibitor to reveal changes in methionine transport between PAS-resistant and sensitive folC unmutated strains (Data not shown). Notably, we also found phenylalanine metabolism might be associated with PAS^r^ resistance in Mtb, however, how it affects PAS resistance needs further study. The results of this study improve our understanding of the mechanisms underlying the response of Mtb to PAS treatment, and may be helpful for the development of novel antibiotics therapy.

## Materials and methods

### Bacterial cultures

Mtb H37Rv strain was cultured on Löwenstein-Jensen (LJ) medium and were treated with PAS according to the route of Figure 1 to obtain PAS-resistance. 218 single colonies were randomly selected and cultured in liquid culture for the extraction of genomic DNA and testing PAS MICs of Mtb strains according to the established protocols [33–35]. Then single colonies were assessed by targeted sequencing of PCR amplicons of loci associated with PAS resistance. The primers used in the targeted sequencing are described in Supplemental Table S8. For downstream experiments, Mtb strains and *M. smegmatis* MC^2^155 were cultured as in Parish and Stoker, 2001 [36] (see Supplemental Experimental Procedures).

### MTT assay

The MTT assay was done as described by Luciano Mengatto et al. [37] (see Supplemental Experimental Procedures).

### Congo red assay

Cells were grown to stationary phase, 2 μL was dropped on 7H10 medium supplemented with 1.5% agar and 100 μg/ml Congo red (Sigma), then incubated at 37°C for 21–30 days [38,39].

### Sliding motility assay

Cells were grown to stationary phase, 2 μl was dispensed on plate containing 7H9 medium without any added carbon source (0.3% agar). The plate was then incubated at 37°C for 21–30 days [38,39].

### Whole genome resequencing

The Mtb genome was extracted as described by Somerville et al. [40]. PCR-free DNA libraries for full-genome sequencing were constructed from the genomic DNA of selected strains using a TruSeq DNA kit (Illumina, Inc.) according to the manufacturer’s protocol, with an average insert size of 350 bp for each sample, and these were assayed using the Illumina MiSeq or HiSeq 2000 sequencing systems. A base-calling pipeline (version HC1.4/RTA1.12) was used to process the raw fluorescent images and call sequences. An average of 520 Mb of data for each sample was generated, representing >100-fold sequence coverage per genome. High-quality paired-end reads were mapped to the reference H37Rv genome (GenBank NC_000962.3) using SOAP2 [41] or Geneious 6.0 (Biomatters Ltd., Auckland, New Zealand) (see Supplemental Experimental Procedures).

### Β-galactosidase assays for determination of promoter activity

Promoters were cloned in pSD5B and the recombinant vectors were used to transform *M. smegmatis* mc_2_ 155 strains. The β-galactosidase activity of the transformants was measured according to standard procedures (see Supplemental Experimental Procedures).

### qRT-PCR

Total RNA was extracted using a FastRNA™ Pro Blue kit (MP Biomedicals, Santa Ana, USA). The cDNA was synthesized from the purified RNA (1 μg) using a SuperScript III First-Strand Synthesis kit (Thermo Fisher Scientific, USA). The mRNA expression of nine different IGR downstream genes relative to *sigA* was determined using the following equation: 2^−(Ct of IGR downstream genes − Ct of *sigA*)^ where Ct is the threshold cycle. This value was used to determine the fold difference in the mRNA expression between PAS^r^ 2 and its parent strain. The fluorescent probe used was SYBR Green (see Supplemental Experimental Procedures).

### Protein preparation and iTRAQ labelling

The proteins were extracted using mechanical crushing method. For each sample, total protein (100 μg) was digested with 3.3 μl of trypsin (1 μg/μl) at 37°C for 24 h. After trypsin digestion, peptides were reconstituted in 0.5 M TEAB and processed according to the manufacturer’s instructions (Applied Biosystems) (see Supplemental Experimental Procedures).

### Liquid chromatography-mass spectrometer (LC–MS) analysis

The resolved peptides were submitted to MS on an Q EXACTIVE (Thermo Fisher Scientific, San Jose, CA, USA) with an analytical C18 column (75 μm i.d. × 150 mm, 2 μm, 100 Å, nanoViper, Thermo Fisher Scientific, USA) for identification and quantification. The raw data were searched with Proteome Discoverer v2.2 version (Thermo Scientific) against the H37Rv database with the same parameters setting as previously described [42]. The final proteins that were deemed to be differentially expressed were filtered as a *P* value <0.05 and 1.5-fold changes (>1.50 or <0.667) relative to the control group.

### Metabolite extraction for LC–MS

The collected Mtb were quenched immediately by liquid nitrogen for 10min stored at −80 °C. The intracellular metabolites in Mtb were extracted according to the method described by Loots du [18] and the metabolites in the culture filtrate were extracted according to the method described by Lau et al. [17]. Quality control (QC) samples were prepared by mixing each aliquot with a pooled sample and analysing them in parallel using the same method. The QCs were injected at regular intervals (every eight samples) throughout the analytical run to provide a set of data from which repeatability could be assessed [17] (see Supplemental Experimental Procedures).

### Identification of metabolites by LC–MS

The samples were analysed in the positive ion mode on an AB 5600 + Triple TOF mass spectrometer system coupled to an Ekspert UltraLC system (110, AB Sciex) which equipped with ACQUITY UPLC HSS T3 (1.8 μm 2.1 × 100 mm, Waters) column. For MS analysis, the capillary voltage was set at +5500 V (positive mode). Other source conditions were kept constant in all the experiments as follows: the pressure of nebulizer gas (nitrogen) was 40 pa. The sheath gas was maintained at a temperature of 550°C. The scan range was adjusted to 100–1200 *m*/*z* (see Supplemental Experimental Procedures).

### Data processing and statistical data analysis

The raw MS files (WIFF format file) were converted to ABF (analysis base file format) using the freely available Reifycs file converter (http://www.reifycs.com/AbfConverter/). Peak picking and alignment were performed using MS-DIAL version 2.24 and the parameters were set as follows: Alignment: MS1 tolerance, 0.01 Da; Retention time tolerance, 0.1 min; Identification: Accurate mass tolerance (MS1), 0.025 Da; Accurate mass tolerance(MS1), 0.25Da [43]. Representative MS/MS spectra were exported in abf format for MS-DIAL, and compound identification was performed against MS/MS libraries including MassBank [44] and MONA [45].

### Bioinformatics analysis

For the proteomics results, Gene ontology (GO) enrichment and Kyoto Encyclopaedia of Genes and Genomes (KEGG) analysis were performed using DAVID online software. For the metabonomics results, multidimensional statistical analysis was performed using MetaboAnalyst software, including unsupervised principal component analysis (PCA), supervised partial least squares discriminant analysis (PLS-DA). Univariate statistical analysis was performed by Students’ t-tests and KEGG pathway analysis was performed using MBROLE 2.0 online software.

## Author contributions statement

W.J.W and T.C conceived, designed and supervised the overall study. L.Z and J.C.Z designed and coordinated the study. H.C.L, H.X.G and Z.Y.L provided the mutated mycobacterial strains. Z.Y.L, J.Z and H.M.Y collected and verified the mycobacterial strains. J.Z, H.M.Y and X.Z.W processed the samples and performed the experiments. W.J.W, H.M.Y, Y.Z and X.L.Z analysed the data. W.J.W and J.Z wrote the paper. All authors read and approved the final manuscript.

## Data Availability

The datasets generated during and/or analysed during the current study are available from the corresponding authors upon request.
